# Detection of circulating tumor cells using manually performed immunocytochemistry (MICC) does not correlate with outcome in patients with early breast cancer – Results of the German SUCCESS-A- trial

**DOI:** 10.1186/s12885-016-2454-3

**Published:** 2016-07-07

**Authors:** Julia Jueckstock, Brigitte Rack, Thomas W. P. Friedl, Christoph Scholz, Julia Steidl, Elisabeth Trapp, Hans Tesch, Helmut Forstbauer, Ralf Lorenz, Mahdi Rezai, Lothar Häberle, Marianna Alunni-Fabbroni, Andreas Schneeweiss, Matthias W. Beckmann, Werner Lichtenegger, Peter A. Fasching, Klaus Pantel, Wolfgang Janni, for the SUCCESS Study Group

**Affiliations:** Department of Gynecology and Obstetrics, Ludwig-Maximilians-University, Munich, Germany; Department of Gynecology and Obstetrics, University Hospital Ulm, Ulm, Germany; Oncology Bethanien, Frankfurt, Germany; Haemotologic-Oncologic Practice Dres, Forstbauer/Ziske, Troisdorf, Germany; Oncologic Practice Dres, Lorenz/Hecker/Wesche, Braunschweig, Germany; Luisenkrankenhaus, Duesseldorf, Germany; Department of Gynecology and Obstetrics, University Erlangen, Erlangen, Germany; University of Heidelberg, Heidelberg, Germany; Charité University Hospital, Berlin, Germany; Institute for Tumor Biology, Hamburg University, Hamburg, Germany

**Keywords:** Breast cancer, Circulating tumor cells, Manual immunocytochemistry, Disease-free survival, Overall survival, Neoplasm, Neoplasm recurrence, Translational research, Detection method

## Abstract

**Background:**

Recently, the prognostic significance of circulating tumor cells (CTCs) in primary breast cancer as assessed using the Food-and-Drug-Administration-approved CellSearch® system has been demonstrated. Here, we evaluated the prognostic relevance of CTCs, as determined using manually performed immunocytochemistry (MICC) in peripheral blood at primary diagnosis, in patients from the prospectively randomized multicenter SUCCESS-A trial (EudraCT2005000490-21).

**Methods:**

We analyzed 23 ml of blood from 1221 patients with node-positive or high risk node-negative breast cancer before adjuvant taxane-based chemotherapy. Cells were separated using a density gradient followed by epithelial cell labeling with the anti-cytokeratin-antibody A45-B/B3, immunohistochemical staining with new fuchsin, and cytospin preparation. All cytospins were screened for CTCs, and the cutoff for positivity was at least one CTC. The prognostic value of CTCs with regard to disease-free survival (DFS), distant disease-free survival (DDFS), breast-cancer-specific survival (BCSS), and overall survival (OS) was assessed using both univariate analyses applying the Kaplan–Meier method and log-rank tests, and using multivariate Cox regressions adjusted for other predictive factors.

**Results:**

In 20.6 % of all patients (*n* = 251) a median of 1 (range, 1–256) CTC was detected, while 79.4 % of the patients (*n* = 970) were negative for CTCs before adjuvant chemotherapy. A pT1 tumor was present in 40.0 % of patients, 4.8 % had G1 grading and 34.6 % were node-negative. There was no association between CTC positivity and tumor stage, nodal status, grading, histological type, hormone receptor status, Her2 status, menopausal status or treatment. Univariate survival analyses based on a median follow-up of 64 months revealed no significant differences between CTC-positive and CTC-negative patients with regard to DFS, DDFS, BCSS, or OS. This was confirmed by fully adjusted multivariate Cox regressions, showing that the presence of CTCs (yes/no) as assessed by MICC did not predict DFS, DDFS, BCSS or OS.

**Conclusions:**

We could not demonstrate prognostic relevance regarding CTCs that were quantified using the MICC method at the time of primary diagnosis in our cohort of early breast cancer patients. Further studies are necessary to evaluate if the presence of CTCs assessed using MICC has prognostic relevance, or can be used for risk stratification and treatment monitoring in adjuvant breast cancer.

**Trial registration:**

The ClinicalTrial.gov registration ID of this prospectively randomized trial is NCT02181101; the (retrospective) registration date was June 2014 (study start date September 2005).

## Background

After having established disseminated tumor cells (DTCs) in the bone marrow as a prognostic factor in metastatic breast cancer [1, 2] in an adjuvant [3–9] and neoadjuvant [10, 11] setting, circulating tumor cells (CTCs) in the peripheral blood of metastatic breast cancer patients have been more recently analyzed with respect to disease-free survival (DFS) and overall survival (OS) by different study groups [12–14]. A European pooled analysis involving 1944 patients with metastatic breast cancer has confirmed the independent prognostic effect of CTCs [15]. CTCs are believed to represent minimal residual disease (MRD) after resection of the primary tumor, with the potential to form distant metastases later in the course of the disease [16, 17]. Accordingly, some recent studies have shown that the presence of CTCs at the time of primary diagnosis is associated with a poor prognosis, that is, reduced DFS as well as shorter OS [18–21]. The independent prognostic value of CTCs (as assessed using the FDA-approved CellSearch® system) in early breast cancer was confirmed in a large pooled analysis of 3173 patients, which showed that both DFS and OS were reduced significantly if CTCs were present at the time of the primary diagnosis [22].

In addition to the CellSearch® system, there are several other techniques available for the detection and enumeration of CTCs [23]; however, data on the prognostic role of CTCs as evaluated using these alternative methods in early breast cancer are lacking, whereas detection of DTCs in bone marrow using cytokeratin-based manual immunocytochemistry (MICC) is well established and has shown prognostic relevance.

The aim of the present study was to evaluate the prognostic relevance of CTCs, as detected by MICC at the time of primary diagnosis, for disease recurrence and survival in a large patient cohort from the SUCCESS-A trial.

## Methods

### Study design

The SUCCESS-A study is a prospectively randomized German multicenter open label phase III trial, investigating the potential benefit of gemcitabine in the adjuvant treatment of primary breast cancer patients. A total of 3754 node-positive or high-risk node-negative patients were randomized to either 3 cycles of FEC followed by 3 cycles of docetaxel or 3 cycles of FEC followed by 3 cycles of docetaxel plus gemcitabine. The study was approved by all involved ethical boards in Germany (reference number 076–05), and written informed consent was obtained from all study participants.

### Patients and procedures

Patient and tumor characteristics were collected for all participants of the trial recruited in 251 German study centers. The tumor stage at primary diagnosis was classified according to the revised AJCC tumor-node-metastasis (TNM) classification [24]. Histopathological grading of the primary tumors was assessed according to the Bloom–Richardson system. Tumors for which immunohistochemical nuclear staining for estrogen, progesterone, or both yielded ≥10 % stained cells were classified as hormone receptor-positive. HER2 positivity was assigned if strong (3+) immunohistochemical membranous staining was present or, in the case of moderate (2+) membranous staining, if an additional fluorescence in situ hybridization (FISH) analysis yielded a positive test result. All patients underwent primary breast surgery (either breast conservation therapy or modified radical mastectomy) leading to R_0_ resection. Routine axillary dissection in patients with positive sentinel lymph nodes included levels I and II. Only when macroscopic metastatic involvement of these lymph nodes was also present, level III lymph nodes were excised. For the diagnosis of lymph node metastasis, single embedded lymph nodes were screened at up to three levels. External beam radiation therapy was administered to all patients treated with breast conserving surgery. Chest wall irradiation following mastectomy was performed in patients with >3 involved lymph nodes or T3 and T4 tumors. After the end of chemotherapy (either FEC-Doc or FEC-Doc/Gemcitabine), premenopausal patients with hormone receptor-positive disease received tamoxifen for 5 years. Endocrine treatment of postmenopausal patients started with tamoxifen for 2 years and was continued with anastrozole for another 3 years.

### Blood sample collection, blood preparation and immunocytochemistry

According to the SUCCESS-A study protocol, blood samples had to be taken from each patient before the start of adjuvant chemotherapy. Usually, CTC detection was performed using the CellSearch® system; however, in cases where too little blood was available for the CellSearch® analysis, or if there was a surplus of blood, CTC detection was performed using the MICC method.

For the study presented here, blood samples (23 ml) from 1221 of the 3754 patients with histologically confirmed invasive primary breast cancer recruited for the SUCCESS-A trial were collected and analyzed using the MICC method; written informed consent was obtained from each patient. If the time span between the collection and preparation of blood samples exceeded 96 h patients were excluded from the analysis. Peripheral blood was collected in tubes containing ethylene diamine tetra acetate (EDTA) and in some cases, also a cell-stabilizing agent (Veridex, Janssen Diagnostics, Raritan, NJ, USA). Samples were shipped at room temperature to the central cancer immunological laboratory at the Women’s Hospital of the Ludwig Maximilians-University of Munich. Blood analyses were performed according to the previously published semi-quantitative assay for bone marrow preparation [25], with the exception of cell enrichment via density gradient centrifugation (OncoQuick® technique), which was performed according to a standard protocol provided by the manufacturer (Greiner BioOne, Frickenhausen, Germany). Here both a liquid separation medium and the subsequent density gradient centrifugation ensured separation of blood cells and granulocytes and specific enrichment of CTCs.

Tumor cell isolation and detection was accomplished based on the Consensus Recommendations [25, 26]. After two centrifugation steps at 500 rpm for 5 min at room temperature, washing (and if needed lysis of red blood cells) and cytospins were prepared by spinning the remaining mononuclear cells onto microscope slides (1,000,000 cells per slide; Menzel, Braunschweig, Germany). The cytospins were air-dried for ≥12 h at room temperature and then used immediately or stored at room temperature.

Immunostaining of cytospins from the blood preparations using the pan-anti-cytokeratin monoclonal antibody A45-B/B3 has been described in detail elsewhere [27]. To detect the specific reaction of the primary antibody, the DAKO- alkaline phosphatase-anti-alkaline phosphatase (APAAP) detection system with the Z0259 antibody serving as a secondary antibody (DakoCytomation, Glostrup, Denmark) combined with new fuchsin staining was used. After capping with cover slips the cytospins were stored at room temperature.

**Fig. 1:**
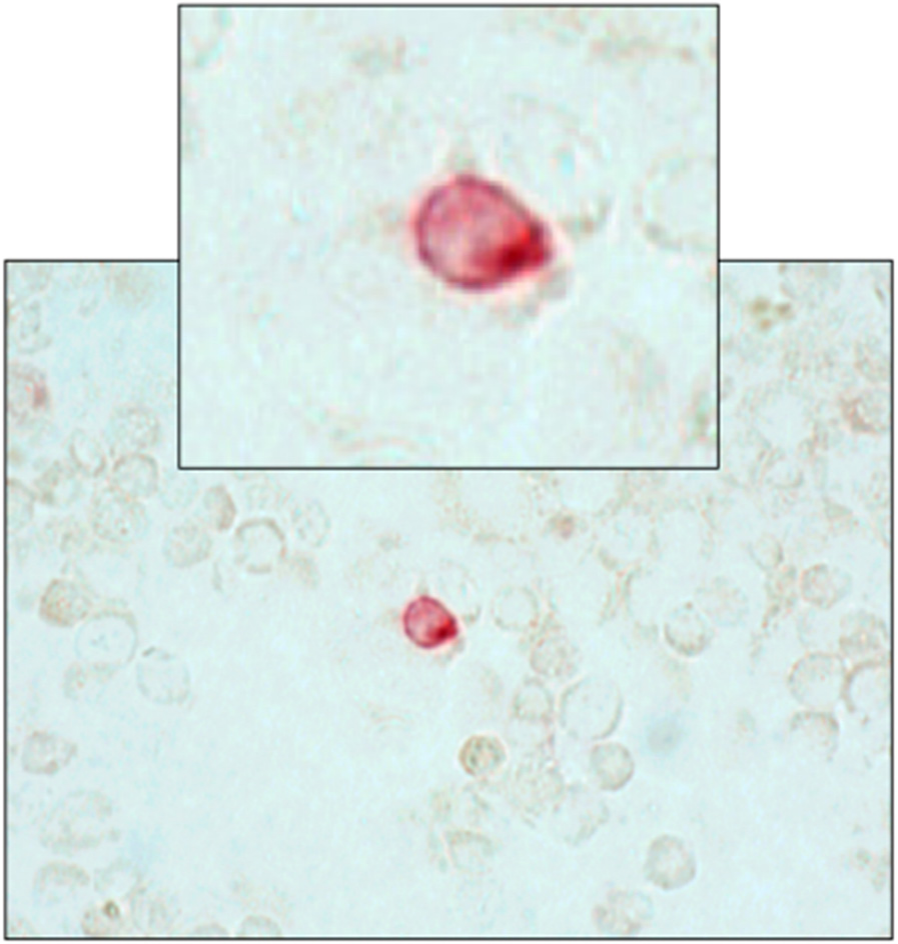
Light-field microscopic image of a circulating tumor cell (CTC) detected using manual immunocytochemistry. Unstained blood cells are visible around the stained CTC

Cytospins containing MCF-7 cells [28] were used as positive controls while cytospins with murine antibody mouse IgG1 kappa (MOPC 21: Sigma, Deisenhofen, Germany) served as negative controls. A total of three cytospins (two cytospins for the detection of CTCs and one negative control) were prepared from all of the blood samples (2 × 10^6^ cells per sample). All cytospins were manually screened for CTCs using conventional light field microscopy (Axiophot: Zeiss, Oberkochen, Germany) or using an automatic device (MDS 1: Applied Imaging Corp., Santa Clara, California, USA) by two independent investigators (Fig. 1). First, cytospins were screened at 20-fold magnification to localize cells suspected of being CTCs; the identity of these cells was then validated by observation at 63-fold magnification. The determination of the presence of CTCs was based on Consensus Criteria, and only immunocytochemically positive cells lacking hematopoietic characteristics, with a moderate to strong staining intensity, were defined as CTCs. Additional criteria for positivity were pathognomonic signs of epithelial tumors, as defined by a clearly enlarged nucleus or clusters of ≥ 2 immunopositive cells [26, 29].

### Statistical analysis

For all categorical variables, descriptive statistics are provided in terms of absolute and relative frequencies. Continuous variables showing data distributions that differed significantly from a normal distribution (as assessed using the Shapiro–Wilk test) are described by reporting medians and ranges. Associations between the presence of CTCs and patients as well as tumor characteristics were analyzed using the following tests: the Mann–Whitney U test for non-normally distributed continuous variables; the Cochran–Armitage test for trends in the ordered categorical variables tumor stage; nodal stage and grading; and the chi-square test for all other categorical variables. These analyses are not part of the primary study objective and have to be interpreted as explorative analyses only; shown are uncorrected *p*-values.

We performed separate analyses for the four survival endpoints, namely OS, BCSS, DFS and distant disease-free survival (DDFS), with the survival endpoints being defined according to the STEEP criteria [30]. Time-to-event data were analyzed using the Kaplan–Meier method and summarized using medians, 95 % confidence limits and Kaplan–Meier survival plots. All time-to-event intervals were measured from the time of the primary diagnosis to the date of the event. If no event was documented, the data were censored at the date of the last adequate follow-up. To assess the simultaneous effects of multiple covariates on the survival endpoints we used Cox proportional-hazards regression models. The initial model included age, tumor stage, nodal stage, tumor grade, histological type, hormone receptor status, HER2 status and menopausal status. We then performed a stepwise backward selection procedure to exclude variables that did not significantly contribute to the model (significance level cutoff for exclusion, 0.10; significance assessed based on the likelihood ratio test), resulting in a final model without the variable CTC presence. In the last step, CTC presence (yes/no) was added to this final model to determine whether or not the inclusion of this variable significantly improved the model, that is, whether the presence of CTCs was a significant independent prognostic factor for survival.

All statistical tests were two-sided, and p values of < 0.05 were considered significant. Statistical analyses were performed using IBM SPSS Statistics, Version 21.0 software (IBM Corp., Armonk, New York, USA).

## Results

### Patient characteristics

The median age of the 1221 patients included in this study was 53 (range, 22–85) years and 42.7 % (*n* = 521) were premenopausal women. More than half of the patients (60.0 %) had tumors >2 cm in size; the vast majority (95.1 %) had grade G2 or G3 tumors, and most of the patients (65.2 %) were node positive. Nearly all of the tumors belonged to the ductal histological subtype (83.5 %), while only 10.0 % of lobular and 6.6 % of other subtypes were found. Overall, 69.7 % of the patients had a hormone receptor-positive tumor, and 24.2 % of the patients showed overexpression of the HER2 gene.

All patients underwent a surgical procedure resulting in R0-resection of their tumor prior to entering the study. Breast conserving surgery was performed in 71.7 % of the patients. In 99.7 % of patients, axillary lymph nodes were excised (in 21.9 % by means of a sentinel lymph node dissection), and only 0.3 % did not receive any axillary staging. All of the patients received adjuvant chemotherapy according to the study protocol. In 85.6 % of the patients, radiation therapy was performed after the end of chemotherapy, and 72.6 % of the patients underwent endocrine treatment. More details regarding patient and tumor characteristics as well as the treatments received are given in Table 1.

**Table 1:** Baseline characteristics of patients and prevalence of circulating tumor cells (CTCs) according to clinicopathological variables

Variable	All patients *N* = 1221	Patients without CTCs *N* = 970	Patients with CTCs *N* = 251	*p*-value^a^
Age (years)				0.41^b^
* Median*	53.0	53.0	54.0	
* Range*	22 - 85	22 - 85	33 - 75	
Tumor stage				0.34^c^
* pT1*	489 (40.0 %)	397 (40.9 %)	92 (36.7 %)	
* pT2*	652 (53.4 %)	511 (52.7 %)	141 (56.2 %)	
* pT3*	63 (5.2 %)	47 (4.8 %)	16 (6.4 %)	
* pT4*	17 (1.4 %)	15 (1.5 %)	2 (0.8 %)	
Nodal stage				0.23^c^
* pN0*	422 (34.6 %)	341 (35.2 %)	81 (32.3 %)	
* pN1*	561 (45.9 %)	446 (46.0 %)	115 (45.8 %)	
* pN2*	165 (13.5 %)	126 (13.0 %)	39 (15.5 %)	
* pN3*	70 (5.7 %)	54 (5.6 %)	16 (6.4 %)	
* unknown*	3 (0.2 %)	3 (0.3 %)	0 (0.0 %)	
Histological grading				0.30^c^
* G1*	59 (4.8 %)	43 (4.4 %)	16 (6.4 %)	
* G2*	604 (49.5 %)	479 (49.4 %)	125 (49.8 %)	
* G3*	557 (45.6 %)	447 (46.1 %)	110 (43.8 %)	
* unknown*	1 (0.1 %)	1 (0.1 %)	0 (0.0 %)	
Histological type				0.26^d^
* ductal*	1019 (83.5 %)	812 (83.7 %)	207 (82.5 %)	
* lobular*	122 (10.0 %)	91 (9.4 %)	31 (12.4 %)	
* other*	80 (6.6 %)	67 (6.9 %)	13 (5.2 %)	
Hormone receptor status				0.73^d^
* negative*	368 (30.1 %)	290 (29.9 %)	78 (31.1 %)	
* positive*	851 (69.7 %)	678 (69.9 %)	173 (68.9 %)	
* unknown*	2 (0.2 %)	2 (0.2 %)	0 (0.0 %)	
HER2 status				0.88^d^
* negative*	906 (74.2 %)	720 (74.2 %)	186 (74.1 %)	
* positive*	296 (24.2 %)	234 (24.1 %)	62 (24.7 %)	
* unknown*	19 (1.6 %)	16 (1.6 %)	3 (1.2 %)	
Menopausal status				0.88^d^
* premenopausal*	521 (42.7 %)	415 (42.8 %)	106 (42.2 %)	
* postmenopausal*	700 (57.3 %)	555 (57.2 %)	145 (57.8 %)	
Type of surgery				0.27^d^
* breast conserving*	876 (71.7 %)	703 (72.5 %)	173 (68.9 %)	
* mastectomy*	345 (28.3 %)	267 (27.5 %)	78 (31.1 %)	
Radiotherapy				1.00^d^
* no*	175 (14.3 %)	139 (14.3 %)	36 (14.3 %)	
* yes*	1045 (85.6 %)	830 (85.6 %)	215 (85.7 %)	
* unknown*	1 (0.1 %)	1 (0.1 %)	0 (0.0 %)	
Endocrine therapy				0.81^d^
* no*	333 (27.3 %)	266 (27.4 %)	67 (26.7 %)	
* yes*	887 (72.6 %)	703 (72.5 %)	184 (73.3 %)	
* unknown*	1 (0.1 %)	1 (0.1 %)	0 (0.0 %)	
HER2-targeted therapy				0.54^d^
* no*	965 (79.0 %)	770 (79.4 %)	195 (77.7 %)	
* yes*	255 (20.9 %)	199 (20.5 %)	56 (22.3 %)	
* unknown*	1 (0.1 %)	1 (0.1 %)	0 (0.0 %)	

^a^All tests without unknowns

^b^Mann–Whitney U test

^c^Cochran–Armitage test for trend

^d^Chi-square test

### Prevalence of CTCs before chemotherapy

**Fig. 2:**
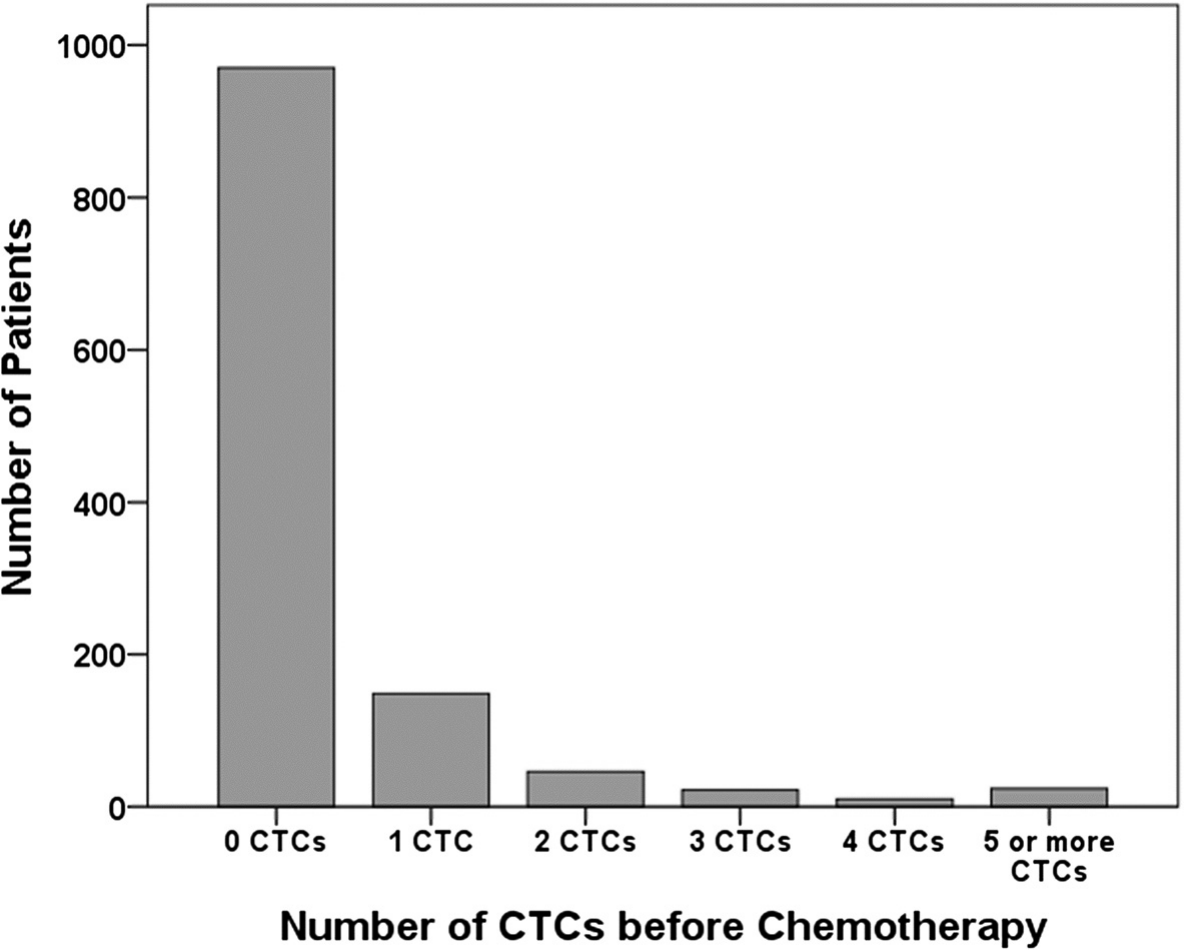
Frequency distribution of the number of circulating tumor cells (CTCs). CTCs were detected using manual immunocytochemistry in the peripheral blood of 1221 patients with early breast cancer at the time of primary diagnosis (before the start of adjuvant chemotherapy)

Collection of peripheral blood was performed not later than 6 weeks after primary diagnosis and R0-resection of the tumor, but always before the commencement of adjuvant chemotherapy. The majority of the patients (*n* = 970; 79.4 %) were negative for CTCs at primary diagnosis. The median number of detected CTCs for the 251 (20.6 %) CTC-positive patients was 1 (range, 1–256) (Fig. 2).

### CTC status according to patient characteristics, tumor biology, and therapy

The presence of CTCs in the peripheral blood after resection of the primary tumor, but before adjuvant chemotherapy, was not significantly associated with patient characteristics (age and menopausal status), tumor characteristics (tumor stage, lymph node status, grading, histological type, hormone receptor status and HER2 status), surgical procedures (breast conserving therapy vs. mastectomy), or therapeutic regimens (Table 1).

### Impact of CTC status on overall and breast-cancer-specific survival

**Fig. 3:**
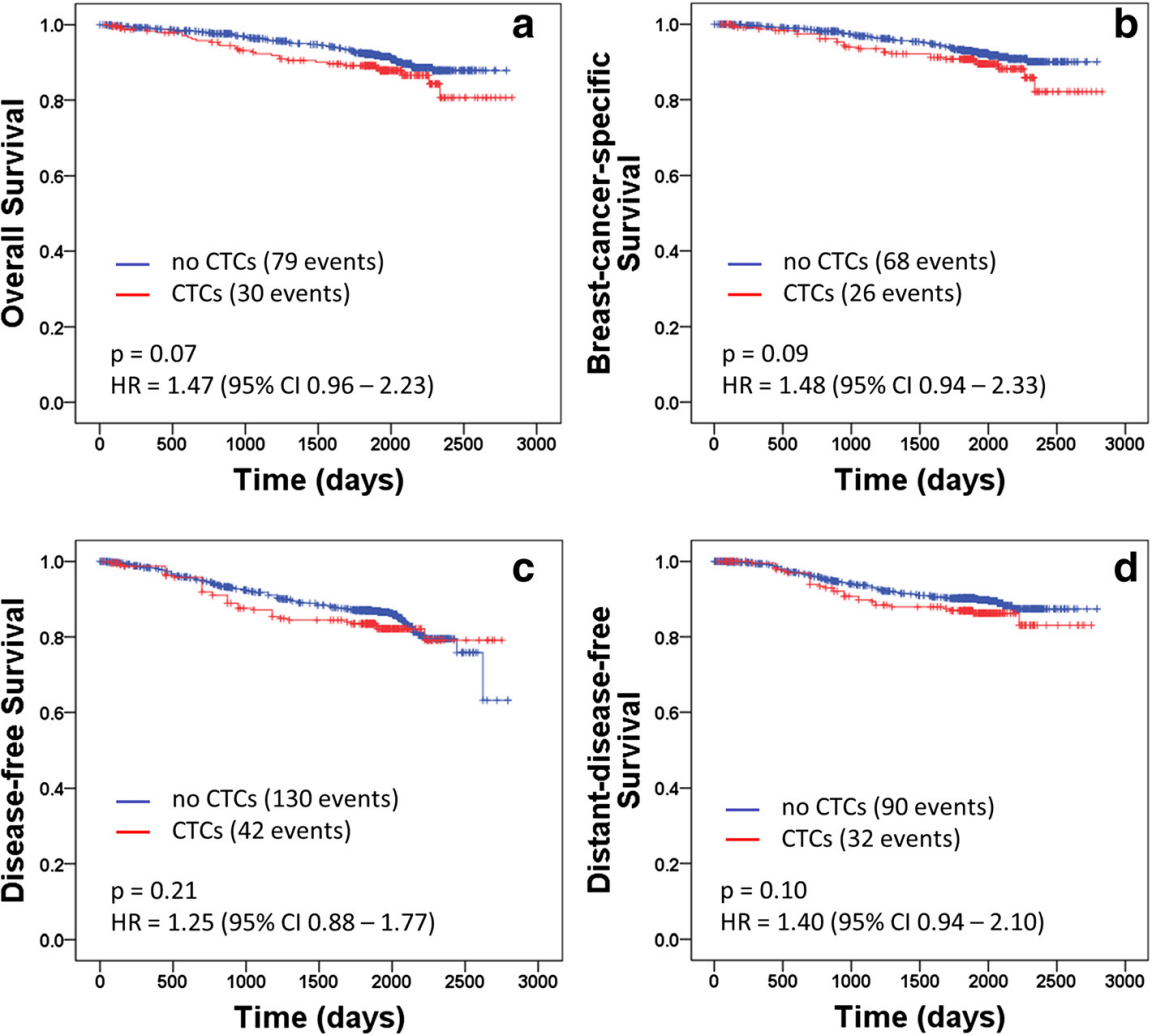
Kaplan–Meier plots of survival. **a** overall survival, **b** breast cancer-specific survival, **c** disease-free survival and **d** distant disease-free survival according to the absence (*n* = 970) or the presence (*n* = 251) of CTCs in the peripheral blood at the time of primary diagnosis. HR denotes the hazard ratio, and p-values refer to log-rank tests

The median follow-up time was 64 months from primary diagnosis. Overall, 109 (8.9 %) out of the 1221 patients died during follow-up, and 94 of the deaths were breast-cancer specific. A total of 79 (8.1 %) of the 970 patients without CTCs at the time of primary diagnosis and 30 (12.0 %) of the 251 patients with CTCs died. Univariate survival analyses revealed a trend for shorter OS in patients with CTCs (hazard ratio (HR), 1.47; 95 % confidence interval (CI), 0.96–2.23; log-rank test, *p* = 0.07; Fig. 3a). Breast-cancer-specific deaths occurred in 68 (7.0 %) patients without CTCs and in 26 (10.4 %) patients with CTCs; similar to OS, univariate survival analysis indicated a trend towards shortened breast-cancer-specific survival in CTC positive patients (HR, 1.48; 95 % CI, 0.94–2.33; log-rank test, *p* = 0.09; Fig. 3b). However, in multivariate analyses, only tumor stage, nodal stage, hormone-receptor status, and HER2 status significantly predicted OS, while the final model for breast-cancer-specific survival additionally included tumor grade (Table 2). The addition of CTC status to the model did not significantly improve model fit (*p* = 0.14 and *p* = 0.17, respectively; Table 2); thus, we could not show that CTC status was an independent prognostic factor for OS or breast-cancer-specific survival.

**Table 2:** Multivariate hazard ratios (HR) for overall, breast cancer-specific, disease-free, and distant disease-free survival

	Overall survival			Breast-cancer-specific survival			Disease-free survival			Distant-disease-free survival		
	HR	95 % CI	*p*-value	HR	95 % CI	*p*-value	HR	95 % CI	*p*-value	HR	95 % CI	*p*-value
Final Model (without CTC presence):												
Age	–	–	–	–	–	–	–	–	–	0.96	0.94 – 0.99	0.007
Tumor stage			<0.001			<0.001			0.001			0.001
* T2 vs. T1*	1.43	0.92 – 2.21	0.11	1.70	1.04 – 2.78	0.03	1.21	0.86 – 1.69	0.27	1.35	0.90 – 2.03	0.15
* T3 vs. T1*	1.97	0.95 – 4.08	0.07	2.78	1.29 – 5.97	0.01	1.35	0.73 – 2.50	0.35	1.22	0.56 – 2.65	0.62
* T4 vs. T1*	9.33	4.01 – 21.69	<0.001	9.98	3.92 – 25.40	<0.001	5.25	2.35 – 11.73	<0.001	6.88	2.62 – 18.06	<0.001
Nodal stage			<0.001			<0.001			<0.001			<0.001
* N1 vs. N0*	1.49	0.91 – 2.45	0.11	1.85	1.08 – 3.18	0.03	1.53	1.03 – 2.27	0.03	2.17	1.32 – 3.57	0.002
* N2 vs. N0*	1.69	0.92 – 3.10	0.09	1.80	0.92 – 3.50	0.09	1.90	1.18 – 3.06	0.01	3.35	1.89 – 5.96	<0.001
* N3 vs. N0*	6.66	3.57 – 12.42	<0.001	8.59	4.42 – 16.68	<0.001	6.65	3.98 – 11.10	<0.001	10.76	5.82 – 19.89	<0.001
Hormone receptor status												
* pos vs. neg*	0.31	0.20 – 0.46	<0.001	0.27	0.16 – 0.43	<0.001	0.35	0.25 – 0.49	<0.001	0.32	0.22 – 0.46	<0.001
Her2 status												
* pos vs. neg*	0.59	0.36 – 0.95	0.03	0.58	0.35 – 0.96	0.04	0.70	0.48 – 1.02	0.06	-	-	-
Grading			-			0.21			-			-
* G2 vs. G1*	-	-	-	4.85	0.66 – 35.49	0.12	-	-	-	-	-	-
* G3 vs. G1*	-	-	-	5.71	0.78 – 41.80	0.09	-	-	-	-	-	-
Menopausal status												
* post vs. pre*	-	-	-	-	-	-	-	-	-	1.66	0.92 – 2.99	0.09
Addition of CTC presence												
CTCs												
* pos vs. neg*	1.38	0.91 – 2.11	0.14	1.39	0.88 – 2.20	0.17	1.19	0.84 – 1.69	0.34	1.34	0.89 – 2.02	0.17

Shown is the final model (Cox proportional hazards regression model, without CTC presence) after backward selection (see text), and the parameter estimates, as well as the significance of the change when CTC presence (yes/no) was added to the model. Please note that the addition of CTC presence did not significantly improve model fit for any of the four analyzed survival endpoints

### Impact of CTC status on disease recurrence

Breast cancer recurred in 172 (14.1 %) patients during the follow-up period, with 130 (13.4 %) relapses in the 970 patients without CTCs and 42 (16.7 %) relapses in the 251 patients with CTCs. Distant metastases occurred in 90 (9.3 %) patients without CTCs and in 32 (12.7 %) patients with CTCs. Univariate analyses showed that CTC status was not significantly associated with DFS (HR, 1.25; 95 % CI, 0.88–1.77; log-rank test, *p* = 0.21; Fig. 3c) or DDFS (HR, 1.40; 95 % CI, 0.94–2.10; log-rank test, *p* = 0.10; Fig. 3d). Multivariate analyses confirmed the lack of significant prognostic relevance regarding CTC presence at the time of primary diagnosis for disease recurrence; this was because the addition of CTC status to the final model obtained after backward selection did not significantly improve model fit, both for DFS (*p* = 0.34) and DDFS (*p* = 0.17). The only significant predictors for DFS in our analyses were tumor stage, nodal stage, hormone-receptor status, and HER2 status, while the final model for DDFS included age, tumor stage, nodal stage, hormone-receptor status, and menopausal status (Table 2).

## Discussion

This is the largest analysis of the prognostic role of CTCs detected using MICC in the setting of a prospectively randomized multicenter trial in early breast cancer patients before adjuvant chemotherapy. However, our investigation did not show an association between the presence of CTCs detected using MICC in the peripheral blood and earlier disease recurrence or reduced survival time. These results are surprising in the context of previous findings obtained in another patient cohort of the same large prospective, randomized multicenter trial (the German SUCCESS-A trial). Using the FDA approved CellSearch® system to assess CTC prevalence at the time of primary diagnosis in 2026 breast cancer patients, Rack et al. demonstrated the presence of CTCs to be a highly significant and independent prognostic factor for OS and DFS with multivariate hazard ratios of 2.18 for OS and 2.11 for DFS [20].

A comparison of CTC prevalence in patients from the SUCCESS-A trial assessed using either the CellSearch® system or MICC revealed that the proportion of patients with CTCs in the peripheral blood at the time of primary diagnosis, as detected using the two methods, did not differ significantly (21.1 % vs. 20.6 %; *p* = 0.75); furthermore, the two patient cohorts were found to be well-balanced with regard to clinicopathological parameters [31]. Thus, it might be expected that the presence of CTCs should be associated with a worse outcome for all patients in the SUCCESS-A study, regardless of the method for CTC detection. Our finding that the presence of CTCs as detected using the MICC method was not significantly associated with reduced survival (in contrast to the presence of CTCs as detected with the CellSearch® system) indicates that the CellSearch® system might be superior to the MICC method in terms of the detection of prognostically relevant CTCs. However, even if the patient cohorts were comparable and well balanced, the two groups were not equivalent and comprised different patients; this hindered a direct comparison between the two methods and interpretation of the results obtained. Head-to-head comparisons between the two methods regarding the same samples could not be performed because of the very small patient number (*n* = 22) for which both CTC detection methods (CellSearch® and MICC) were used. Thus, there could be other as yet unidentified differences between the two patient cohorts that account for the fact that the presence of CTCs was associated with a significantly poorer prognosis in patients where CTC prevalence was determined using the CellSearch® system, but not in patients where CTC prevalence was determined using the MICC method. Other possible explanations for the differing results are pre-analytical factors caused by the fact that for the MICC method patients were chosen from whom too little blood was available to perform the CellSearch® analysis. The fact that we found no significant association between the presence of CTCs detected using the MICC method and survival, was not the result of a lack of statistical power; a retrospective power analysis showed that our study had 95 % power (two-sided, alpha 0.05) in the detection of a DFS hazard ratio of 2.0 for patients with CTCs relative to patients without CTCs. For comparison, the univariate DFS hazard ratio for the SUCCESS-A patients with CTCs detected using the CellSearch® system, as compared with patients without CTCs as reported by Rack et al. [20], was 2.26. Thus, we are confident that our study was sufficiently powered to detect a prognostic value of CTCs assuming a hazard ratio similar to the one reported by Rack et al. [20]. In our view, the most probable explanation for the incongruent findings of the current study as compared with the previous analysis [20] is that the two methods differ with regard to the subpopulations of circulating cells that are detected, even if we cannot completely exclude the possibility that the observed differences are caused by an unidentified bias between the two patient cohorts.

Both the CellSearch® system and the MICC technique are based on labeling of cytokeratin-containing cells (and therefore epithelial cells) in peripheral blood [32]. Using the CellSearch® system CTCs are separated from blood cells on the basis of epithelial cell adhesion molecule (EpCam) expression, while in the MICC method this separation is achieved using a density gradient centrifugation. Former investigations in healthy individuals and in patients with benign tumors as compared with patients with malignant tumors [33] have shown the high specificity of the CellSearch® system in reliably detecting malignant epithelial cells. The carcinomatous origin of the detected CTCs using the MICC method has also been proven [34]. One difference between the two techniques is that the CellSearch® system is a semi-automated method while the MICC technique is performed manually. Consequently, the MICC technique is the more elaborate and time-consuming process; as a manual technique it has a higher intertest variability concerning CTC detection in peripheral blood. However, the final decision as to whether or not a cell is considered a CTC using both techniques has to be made by an investigator, and cannot be carried out using an automated method. Another important difference is that the MICC method does not have a CD 45 counterstaining step for the separation of leukocytes from CTCs; thus, it possibly yields a higher amount of prognostically less relevant cells (such as leukocytes) that impair the prognostic value of this method. In brief, the main differences between MICC and the CellSearch® are as follows: the cell enrichment is achieved via density gradient centrifugation using MICC and with an automated cell enrichment method using the CellSearch® system; the APAAP technique is applied for antibody staining using MICC and immunomagnetic antibody enrichment using CellSearch® (however, both methods use the same antibodies directed against cytokeratins CK8, CK18, and CK19); and the lack of CD 45 counterstaining in the MICC method. Thus, it seems possible that some of the observed inconsistent findings with regard to CTCs detected with the CellSearch® system or the MICC method might be related to technical details or procedures.

In bone marrow analyses concerning DTCs, the MICC method is considered the gold standard because of its high reliability and reproducibility [25]. However, with respect to the detection of CTCs in the peripheral blood, the CellSearch® system seems to be more reliable and represents the most established method, especially with regard to the evaluation of the prognostic value of CTCs. There have been some studies that have compared the CellSearch® system with other methods for CTC detection and enumeration. In a direct comparison based on blood samples collected from 61 patients with metastatic and non-metastatic cancer, as well as 15 healthy donors, the CellSearch® system proved to be more sensitive than a manual detection technique using OncoQuick®. CTCs were detected in 33 (54 %) out of the 61 cancer patients using the CellSearch® system, but in only 14 (23 %) of these patients using OncoQuick® [35]. Interestingly, considering only patients with non-metastatic cancer, the detection rate of CTCs was equal using both methods (12 %); it was considerably lower than the detection rate found in our study (20.6 %). However, it should be noted that the staining process (DAPI, Alexa Fluor 555) used in the study by Balic et al. [35] differs from the staining method (CK, new fuchsin) used in our analysis. Using another fluorescence based detection method Pachmann et al. also demonstrated the prognostic relevance of CTCs in primary breast cancer patients [36]. A study comparing three different CTC detection methods in metastatic breast cancer patients found that a molecular technique based on a combined quantitative reverse-transcription polymerase chain reaction (qRT-PCR) approach for CK-19 and mammaglobin was more sensitive than the CellSearch® system or the AdnaTest BreastCancer®, which is another commercially available CTC assay based on the detection of three tumor-associated transcripts (GA733-2, MUC-1, and HER2) using the reverse transcription-polymerase chain reaction (RT-PCR) after immunomagnetic enrichment of tumor cells [37]. Two more studies compared the AdnaTest BreastCancer® with the CellSearch® system in patients with metastatic breast cancer. One study revealed a high concordance between the AdnaTest BreastCancer® and the CellSearch® system in the detection of two or more CTCs without evaluating the prognostic relevance [38]. In contrast, the prospective multicenter German DETECT study showed a higher positivity rate for CTCs using the CellSearch® System (cutoff level ≥5 CTCs; 122 out of 245; 50 %) compared to the AdnaTest BreastCancer® (88 out of 221; 40 %) [39]. Furthermore, CTC positivity assessed based on the CellSearch® system was found to be a significant prognostic factor for OS in both univariate and multivariate analyses (HR, 2.7; 95 % CI, 1.6–4.2; *p* < 0.01), while CTC-positivity assessed using the AdnaTest BreastCancer® had no significant association with progression-free survival (PFS) or OS [39]. In summary, in comparing different CTC detection methods, the FDA approved CellSearch® system seems to be one of the most reliable techniques; however, the MICC method is still considered the gold standard regarding DTC detection in the bone marrow.

## Conclusions

To our knowledge, no direct comparison of different manual detection methods for CTCs has been previously reported; thus, until now no conclusion can be drawn as to which of the manually performed CTC analyses is the most reliable method. Here, we could not demonstrate the prognostic relevance of CTCs detected using MICC in early breast cancer patients. This contrasts with the findings of Rack et al. [20] and Lucci et al. [21], who reported that CTC positivity as assessed using the CellSearch® system was significantly associated with reduced PFS and OS. Our results show that the presence of CTCs as assessed using manual detection methods is not associated with survival; they also indicate that, although not necessarily the most sensitive technique, the CellSearch® system seems to be the more reliable method concerning the detection of prognostically relevant CTCs in early and metastatic breast cancer.

## Abbreviations

APAAP, alkaline phosphatase-antialkaline phosphatase; BCSS, breast-cancer-specific survival; CI, confidence interval; CK, cytokeratin; CTC, circulating tumor cells; DDFS, distant-disease-free survival; DFS, disease-free survival; DTC, disseminated tumor cells; EDTA, ethylene diamine tetra acetate; EpCam, epithelial cell adhesion molecule; FDA, food and drug administration; FISH, fluorescence in situ hybridization; HR, hazard ratio; MICC, manually immunocytochemistry; MRD, minimal residual disease; OS, overall survival; qRT-PCR, quantitative real time polymerase chain reaction; SUCCESS, Simultaneous Study of Gemcitabine-Docetaxel Combination adjuvant treatment, as well as Extended Bisphosphonate and Surveillance.
